# *LibraEDT*: a software solution for automated 3D-ED data acquisition

**DOI:** 10.1107/S1600576725006892

**Published:** 2025-09-04

**Authors:** Moussa D. Faye Diouf, Mauro Gemmi

**Affiliations:** a Electron Crystallography, Istituto Italiano di Tecnologia, Pontedera56025, Italy; b Department of Chemical, Life and Environmental Sustainability Sciences, University of Parma, Parma43123, Italy; SLAC National Accelerator Laboratory, Menlo Park, USA

**Keywords:** crystal tracking, electron microscopy, three-dimensional electron diffraction, 3D-ED

## Abstract

The *LibraEDT* software has been developed to enhance and automate 3D electron diffraction (3D-ED) experiments by addressing key challenges such as crystal tracking, low-fluence data acquisition and better data management. This tool significantly improves both the accuracy and efficiency of ED workflows, particularly when applied to beam-sensitive materials. The utility of this approach is demonstrated through the structural analysis of a known beam-sensitive organic crystal, as well as a novel and previously unreported zinc-based metal–organic framework incorporating protocatechuic acid.

## Abstract 2

We present *LibraEDT*, a program that automates and optimizes 3D electron diffraction experiments. This approach allows the accurate structure determination of materials such as beam-sensitive organic crystals and metal–organic frameworks. The current implementation was developed to optimize experiments on the Zeiss Libra 120 kV microscope and Timepix1 detector, but the algorithms and proposed workflow can be adapted to other setups.

## 1. Introduction

Crystallography is a fundamental discipline, offering un­paralleled insights into the intricate atomic arrangements that determine the properties and behaviours of diverse materials. Characterizing nanocrystals presents unique challenges due to their small size, which limits the applicability of traditional crystallographic techniques. Single-crystal X-ray diffraction (SCXRD), the most widely used method for crystal structure determination, becomes increasingly difficult to employ as the crystal size decreases below approximately 1 µm. This is primarily because smaller crystals produce weaker diffraction signals owing to the loss of coherence, and these signals are often insufficient for accurate data collection and structure determination. Similarly, powder X-ray diffraction (PXRD), a valuable tool for analysing crystallinity and phase identification in nanocrystalline materials, can struggle with structural solutions, particularly when dealing with complex or heterogeneous samples. This difficulty arises because PXRD aggregates diffraction data from numerous crystallites, leading to overlapping diffraction peaks due to the one-dimensional nature of the data. This problem is intensified if the sample is heterogeneous, as different phases or minor components contribute additional peaks, making it challenging to distinguish between closely related phases or to model the structure accurately.

To address these challenges, electron diffraction (Kolb *et al.*, 2007 ▸; Kolb *et al.*, 2008 ▸) (ED) has emerged as a powerful technique specifically suited for studying nanocrystals. Electrons are much stronger scatterers (Bethe, 1928 ▸; Vajnštejn, 1956 ▸) than X-rays, allowing ED to produce detectable diffraction signals from smaller crystals. By directing an electron beam at a single-crystalline grain, a single-crystal diffraction experiment can be performed. The collected data can then be analysed to reveal detailed information about the crystal’s atomic arrangement.

Within the scope of ED, several specialized methods exist under the umbrella of 3D electron diffraction (3D-ED) (Gemmi *et al.*, 2019 ▸). These methods include automated diffraction tomography (Kolb *et al.*, 2007 ▸; Kolb *et al.*, 2008 ▸), precession electron diffraction tomography (PEDT) (Mug­naioli *et al.*, 2009 ▸), continuous rotation electron diffraction (cRED) (Nederlof *et al.*, 2013 ▸; Gemmi *et al.*, 2015 ▸), microcrystal electron diffraction (Nannenga *et al.*, 2014 ▸) and rotation electron diffraction (Zhang *et al.*, 2010 ▸).

While all these 3D-ED methods enable the study of crystals too small for conventional SCXRD (Sala *et al.*, 2024 ▸; Marchetti *et al.*, 2023 ▸), cRED has become particularly popular owing to its simplicity and the fact that the sample can be rotated continuously during data collection, analogously to SCXRD with area detectors adapted to the electron microscope environment. However, cRED experiments pose several challenges. Maintaining the sample’s position within the electron beam during continuous rotation can be problematic because of goniometer instabilities and stage imprecision. These issues can lead to the crystal drifting out of the illuminated area, complicating the data collection, increasing the experiment duration and exposing beam-sensitive samples to unnecessary radiation, potentially damaging the material under study. Additionally, preparing input files for data processing can be time consuming, especially when data acquisition is interrupted due to the crystal moving out of the illumination area. In these situations, the experiment must resume from the last recorded angle, and the resulting diffraction patterns from each session need to be manually merged into a complete data set.

To tackle all these issues, this paper presents a software solution, *LibraEDT*, developed mainly in C++, specifically designed to optimize and automate 3D-ED experiments conducted on the Zeiss Libra 120 kV microscope. The software addresses the challenges faced during experiments by integrating advanced control over the microscope’s components, ensuring consistent crystal positioning and automating manual data management tasks. Not only does this speed up the workflow but it also reduces the potential for human error which can arise from manual data entry. This is particularly important when handling large data sets, as it ensures consistency in data processing and reduces the potential for errors. Moreover, the software facilitates high-throughput experiments, allowing for the rapid collection of large amounts of data in a short amount of time. This is a crucial step toward automated large-scale ED studies in the future, where high-quality data can be acquired with minimal human intervention.

## 2. Software implementation

Although the *LibraEDT* software was developed to optimize cRED experiments on the Zeiss Libra 120 kV microscope, the algorithms and proposed workflow can be adapted to any microscope. Focusing on addressing specific challenges, the software aims to ensure crystal permanence within the illuminated area, to automate manual data preparation, and to provide precise control and reproducibility of the experimental setup, whether it is operated in conventional transmission electron microscope (TEM) mode or in scanning transmission electron microscope (STEM) mode.

**Figure 1:**
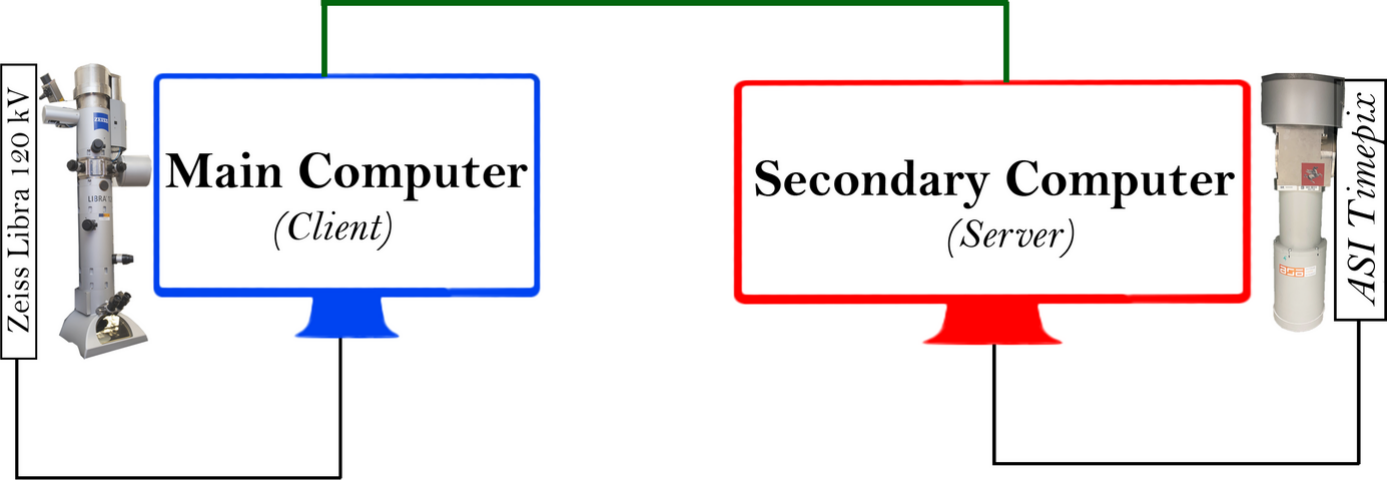
Schematic representation of the experimental setup. The main computer (client) controls the Zeiss Libra 120 kV TEM, while the secondary computer (server) handles data acquisition from the ASI Timepix detector. The two computers are networked to enable data transfer and synchronized control of the experiment.

The design of *LibraEDT* is based on a modular architecture, which was necessary due to the separate installation of the TEM and the ASI Timepix hybrid pixel detector. On one side, the TEM, equipped with an omega filter, is connected to and controlled by a main computer (client) running Microsoft Windows 7, which manages all microscope-related operations such as stage control, beam alignment and lens configurations. On the other side, the ASI Timepix detector is connected to a secondary computer (server) running Ubuntu Linux, responsible for data acquisition from the detector. This separation required the development of two different modules: the Server Module and the Client Module. Each module is designed to control and communicate with specific hardware components, ensuring efficient and reliable operation during 3D-ED experiments. Our experimental setup is illustrated in Fig. 1 ▸.

### 2.1. Server Module: advanced data capture

The Server Module, implemented in Python, operates on the secondary computer directly connected to the ASI Timepix detector. Its primary function is to manage data acquisition by processing requests from the Client Module, such as adjusting exposure times and acquiring single or multiple frames. To execute these commands, the Server Module loads and uses a custom C++ library specifically designed to initialize and control the detector. Running these tasks on the secondary computer drastically reduces the load on the main TEM computer, therefore improving the system’s responsiveness.

To the best of our knowledge, this implementation represents the first publicly available server specifically developed for the ASI Timepix1 detector. While newer versions of the detector, such as the Timepix3 and Medipix3, are equipped with built-in server capabilities and a toolkit known as *Serval*, which allows similar control and manipulation of the detector, the original Timepix1 lacked this functionality. *LibraEDT*’s Server Module addresses this gap by providing a robust solution for managing the Timepix1 detector, enabling it to perform functions typically available only in more recent hardware iterations.

### 2.2. Client Module: comprehensive control and live imaging

The Client Module, entirely developed in C++, serves as the core component of *LibraEDT*, managing the interaction between the TEM and the ASI Timepix detector. On one hand, it communicates directly with the TEM through its application programming interface (API), facilitating comprehensive manipulation and control of the microscope’s functionalities. This includes stage control, lens and coil manipulation, and overall microscope configuration, allowing for detailed control over the experimental setup.

On the other hand, the Client Module interfaces with the Server Module via a TCP/IP connection, enabling remote control of the detector. This communication channel allows the Client Module to send different command requests to the server. Additionally, the Client Module receives image data from the Server Module, which are processed and displayed in real time as a live visualization, providing the operator with immediate feedback on the specimen or diffraction patterns.

**Figure 2:**
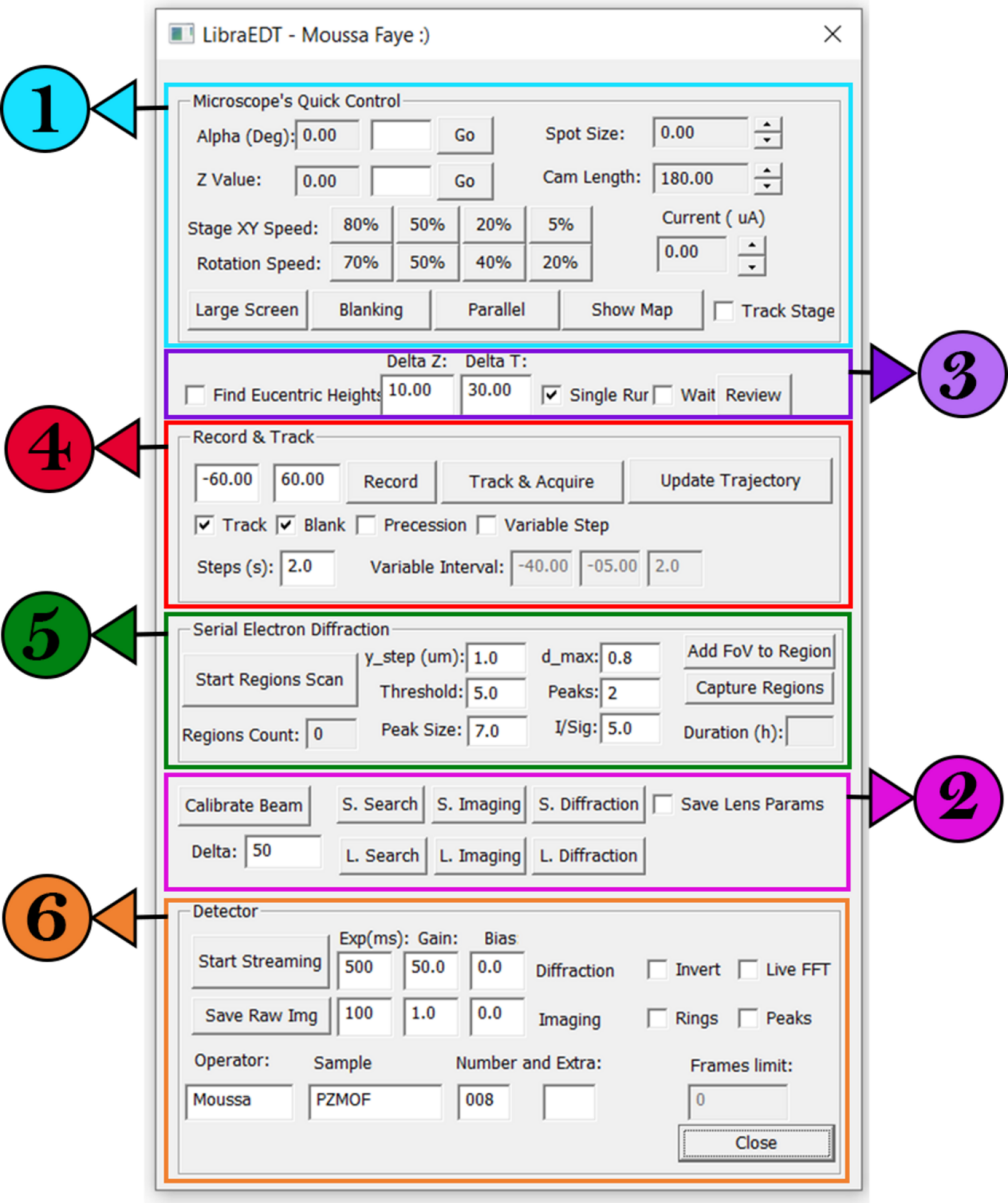
GUI of the *LibraEDT* Client Module with highlighted sections, illustrating the main operational areas as discussed in the text: the numbered circles refer to subsections in Section 3 where the functionality of each is described in detail.

All these functionalities are integrated into a user-friendly graphical user interface (GUI), as illustrated in Fig. 2 ▸. The intuitive design of the GUI ensures that users can easily access and manage the complex control options without requiring extensive technical expertise. *LibraEDT* unifies the control of both the TEM and the detector into a single software platform, running on a single computer. This integration makes the experimental workflow much easier and more straightforward, enhancing the overall efficiency and reliability of 3D-ED experiments.

## 3. Core functionalities

In the following subsections, we will provide a detailed explanation of the software’s core functionalities, focusing in turn on each of the different sections highlighted and numbered in Fig. 2 ▸.

### 3.1. ‘Microscope’s Quick Control’

The section numbered 1 in Fig. 2 ▸ provides access to essential functionalities for 3D-ED experiments, with many controls useful for beam-sensitive samples. For example, tuning the rotation speed helps protect delicate materials by reducing their exposure time to the electron beam. Adjustments of the spot size and filament current allow precise control over the electron flux, while the beam blanking option turns off the beam during idle periods to minimize sample damage. Additional controls include camera length adjustment for optimizing diffraction resolution, and the ability to manage stage position and rotation. When operating *LibraEDT* in STEM mode, users can switch between parallel and convergent-beam modes by relaxing and exciting the C3 lens, respectively.

### 3.2. Beam calibration and operational settings

#### 3.2.1. Beam calibration

The ‘Calibrate Beam’ button (section 2 in Fig. 2 ▸) is essential for precise control of the electron beam’s position through the image visualized by the detector. This feature determines the number of lens current adjustments needed to shift the electron beam by a specific number of pixels in the displayed image, effectively mapping the physical beam movements onto the digital interface.

**Figure 3:**
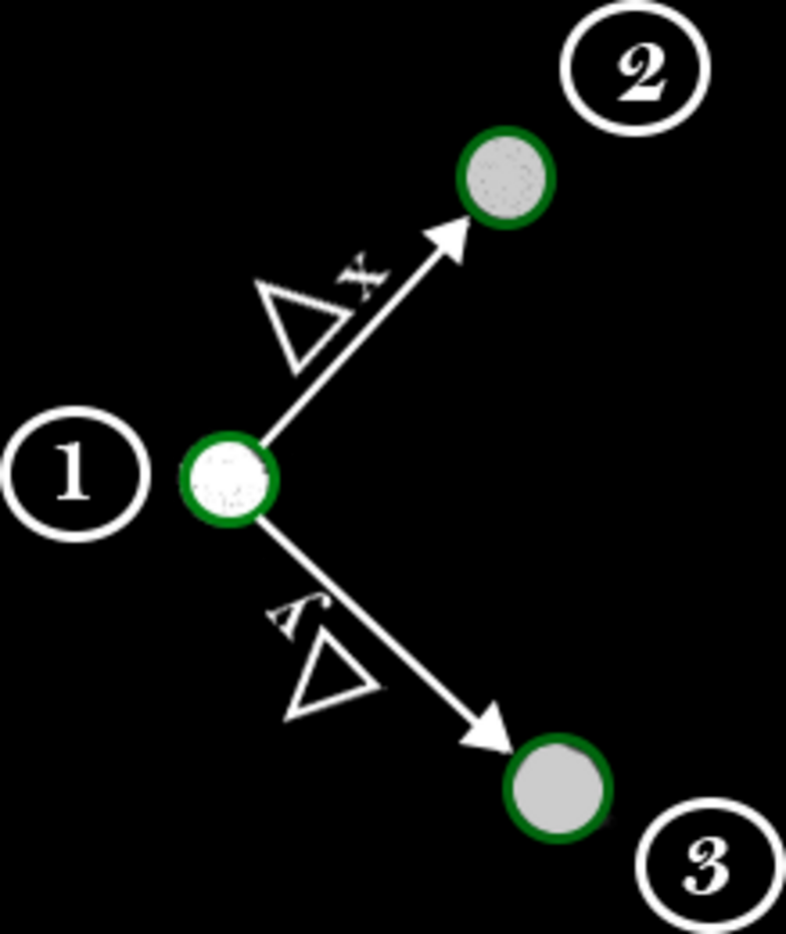
Superimposition of the three beam images captured during the calibration process. Labels refer to the reference position (1) and the shifted positions along the *x* axis (2) and *y* axis (3).

The calibration process, which is a different and simpler approach to the one proposed by Smeets *et al.* (2018 ▸), involves capturing three parallel beam images: one at the initial position as a reference, a second after shifting the beam along the *x* axis and a third after shifting it along the *y* axis (Fig. 3 ▸). Afterwards, the user selects the beam’s size and position in the reference image, followed by the shifted positions in the subsequent images. These inputs are used to calculate the beam shift vectors in the *x* and *y* directions, determining how much the beam moves per unit of current in the lenses. The current values for these shifts are defined in the GUI’s ‘Delta’ textbox.

This calibration can also be automated in *LibraEDT* and, once complete, it allows users to direct the beam to any point on the screen by simply clicking on the desired location in the image, as illustrated later (Fig. 7).

#### 3.2.2. Operational settings

The ‘S. Search’, ‘S. Imaging’ and ‘S. Diffraction’ buttons in section 2 in Fig. 2 ▸ allow the operator to store different condenser aperture and lens settings for various operational modes. This functionality simplifies the process of maintaining different electron dosage levels for crystal searching, imaging and diffraction data acquisition. For example, low electron fluences can be used for searching and imaging to protect beam-sensitive samples, while higher fluences can be applied during diffraction to achieve a better signal-to-noise ratio.

The corresponding settings can be loaded at any time using the ‘L. Search’, ‘L. Imaging’ and ‘L. Diffraction’ buttons. How­ever, *LibraEDT* will automatically load these settings when appropriate. For instance, diffraction settings are loaded during diffraction quality checks or data acquisition, while imaging settings are applied when capturing and saving images to disk. Note that the Zeiss Libra is a three-condenser system operating under Köhler illumination (Benner & Probst, 1994 ▸), in which the illuminated area on the sample is set exclusively by the selected condenser aperture rather than by the excitation of the final condenser lens. As a result, beam intensity can be adjusted via lens currents without affecting the beam diameter, unlike most TEMs, giving fully parallel illumination ideal for both imaging and diffraction.

**Figure 4:**
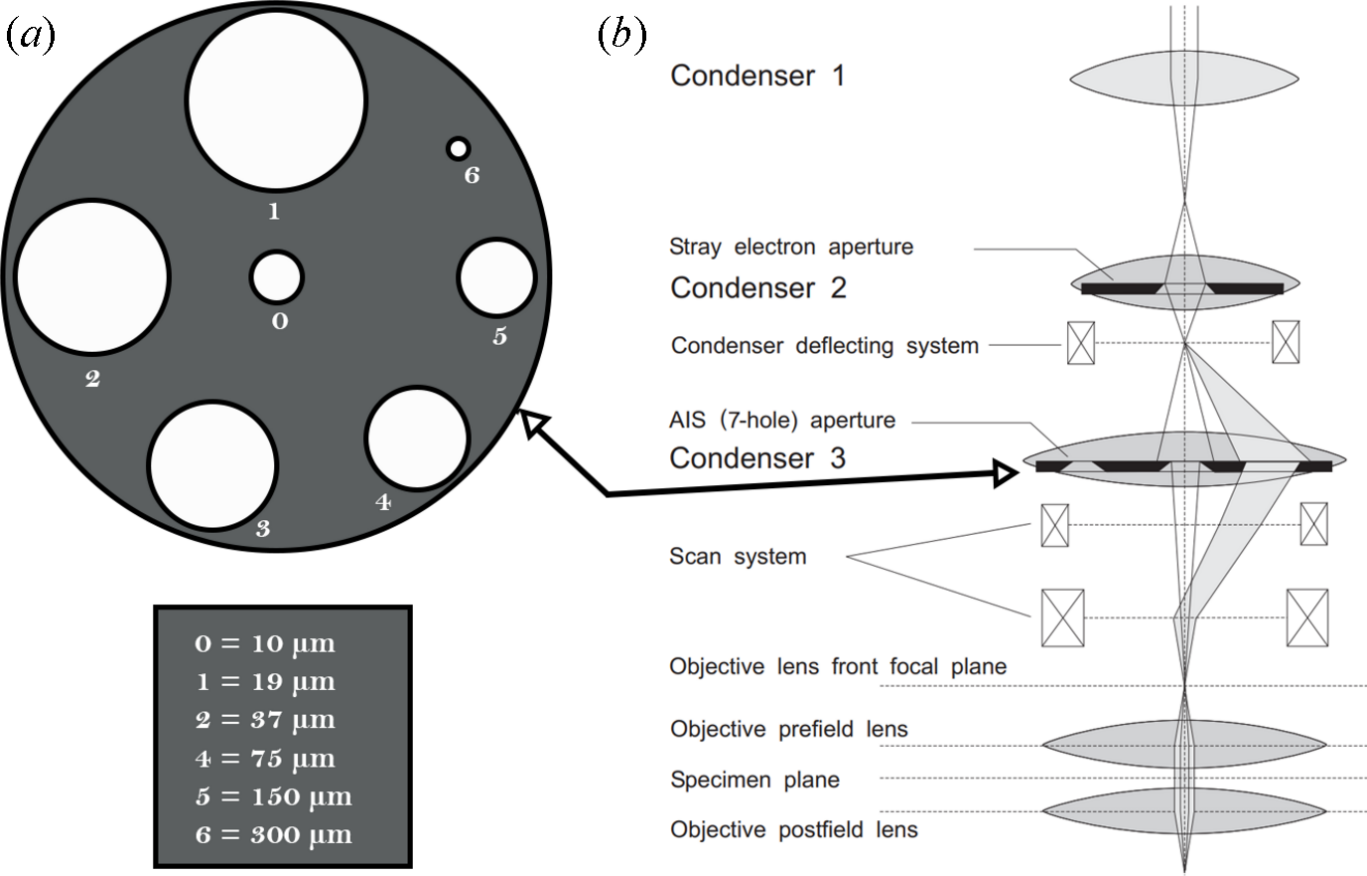
(*a*) Multi-hole condenser aperture with corresponding sizes, and (*b*) schematic representation of the AIS in Zeiss microscopes.

To take advantage of this capability, we make use of the automatic illumination-aperture selection (AIS) system. This system incorporates a condenser deflecting system that electronically shifts the electron beam to any of the desired apertures [Fig. 4 ▸(*a*)] and a scanning system, located below the condenser aperture, that compensates for this shift, returning the beam back to the optical axis [Fig. 4 ▸(*b*)]. As a result, we can use a multi-hole condenser aperture [Fig. 4 ▸(*a*)] (with seven apertures for our setup) to define the size of the illuminated area, without the need for motorized apertures or microscope realignment.

### 3.3. Automatic eucentric height calculation

Adjusting the eucentric height is a critical step in 3D-ED experiments to ensure that the crystal remains within the illuminated area during stage tilting by minimizing lateral specimen movement. While several methods exist for estimating the eucentric height, a commonly used approach involves tilting the electron beam away from the optical axis and minimizing the observed shift of the crystal. This beam-tilt method is fast but often imprecise.

In *LibraEDT*, the eucentric height is calculated using a more accurate automated method, closely mirroring the manual adjustment process (Koster *et al.*, 1992 ▸; Ziese *et al.*, 2002 ▸). By acquiring images at two distinct tilt angles, α_1_ and α_2_, as well as at two different stage heights, *z*_1_ and *z*_2_, the displacement of the crystal *d* can be characterized as a function of both tilt and height. These measurements enable a linear regression approach that isolates the influence of height adjustments on the observed displacement.

At each tilt angle, the crystal displacement *d* is measured at both heights. From these data, the change in displacement at *z*_1_ is 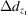 and the change in displacement at *z*_2_ is 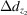. Their difference, 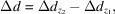
together with the known height difference, Δ*z* = *z*_2_ − *z*_1_, is used to calculate the slope (*m*) of the linear regression,
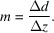
The intercept (*c*) is determined from
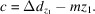
Finally, the optimal eucentric height *z*_euc_ is found by
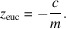


This approach results in better eucentric height estimation. However, it is slower than the beam tilt approach and is therefore not commonly used in our routine workflows. Nevertheless, having this feature available in *LibraEDT* is advantageous, particularly for potential future applications such as unsupervised data acquisition.

### 3.4. Crystal tracking

**Figure 5:**
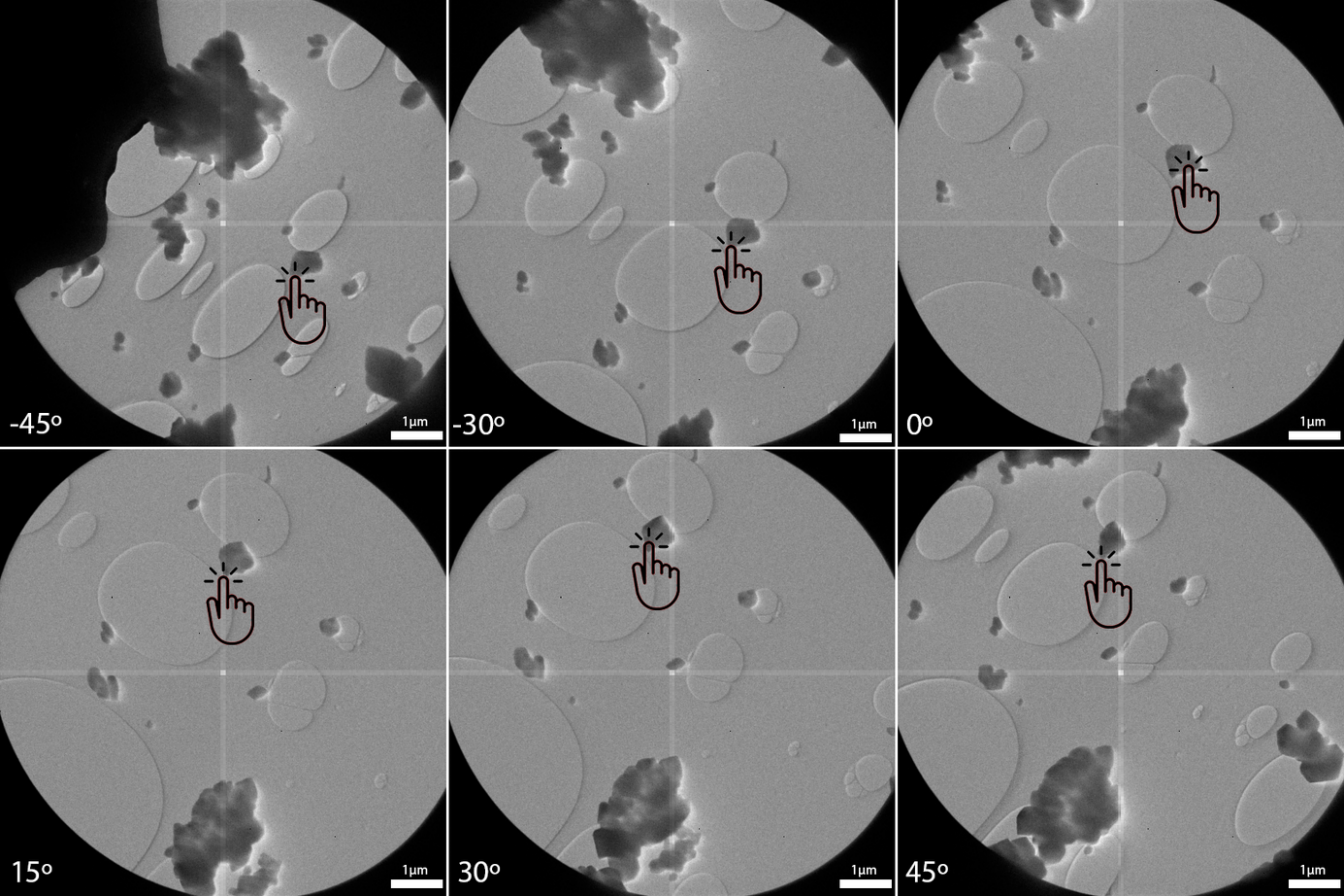
Illustration of how the crystal’s position is marked by the user (hand icon) in images acquired at different tilt angles to generate a trajectory.

Crystal tracking is one of the most important functionalities offered by *LibraEDT* and is implemented in section 4 of the interface. In contrast to the approach used by *Instamatic* (Cichocka *et al.*, 2018 ▸), where tracking relies on periodically defocusing the diffraction pattern via the intermediate lens (IL1) and manually shifting the stage, *LibraEDT* first records the crystal’s trajectory during stage rotation and then continuously adjusts the beam position to ensure that the crystal remains within the beam throughout diffraction data collection. This method was first proposed by Gemmi *et al.* (2015 ▸) and later implemented by Plana-Ruiz *et al.* (2020 ▸). The process can be performed in both STEM and TEM modes and begins by selecting the ‘Record’ option, which loads previously defined imaging parameters (section 2 of the interface, Section 3.2.2) and initiates stage rotation from the specified initial angle to the final angle. Images are acquired at user-defined time intervals and, when the recording is finished, they are displayed to the user, who marks the crystal’s position in each frame (Fig. 5 ▸). This manual selection of the beam position allows for data acquisition on different regions of the crystal, reducing quality loss due to beam damage. While the user is marking the positions, the stage automatically rotates back to the initial angle in preparation for subsequent procedures. This step results in a trajectory map describing the crystal’s positional changes over the course of the rotation. If the ‘Blank’ checkbox is selected, the beam remains blanked at all times except for the brief moments when images are captured.

Once this trajectory is established, the ‘Track & Acquire’ option is selected, causing *LibraEDT* to switch into diffraction mode and load the previously configured diffraction settings (section 2 of the interface, Section 3.2.2). The stage is then rotated through the same angular range, while the software continuously adjusts the electron beam position on the basis of the determined trajectory to keep it centred on the crystal. To achieve this beam adjustment, the trajectory can be interpolated using either linear or spline methods. Although linear interpolation is simpler and often sufficient, spline inter­polation is generally preferred as it provides a smoother path. In most cases, the difference between the two approaches is negligible. However, spline interpolation becomes particularly beneficial if the crystal undergoes abrupt changes in direction. These precise adjustments are further enabled by the prior beam calibration step (section 2 of the interface, Section 3.2.1), which correlates deflection coil currents with the corresponding screen coordinates.

This approach is quite versatile as it allows for data acquisition in both STEM and TEM modes. *LibraEDT* also supports PEDT via the dedicated ‘Precession’ checkbox. Enabling this option switches the software to the conventional stepwise PEDT workflow, pausing at each tilt increment to perform the precession sequence.

**Figure 6:**
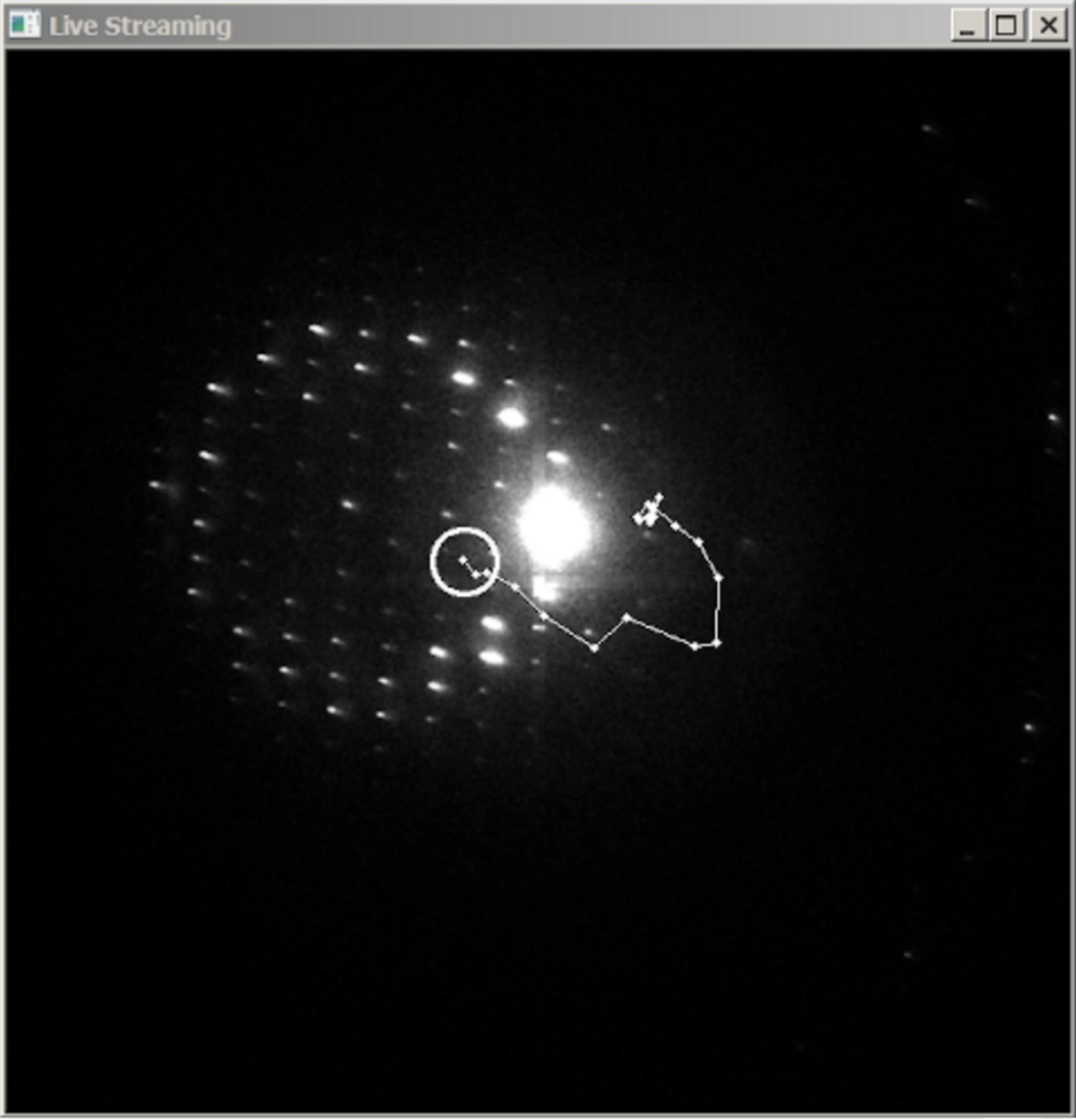
Example of the visual overlay of the crystal trajectory and a virtual beam marker during the ‘Track & Acquire’ process.

During data acquisition, real-time visual feedback is provided by superimposing the generated crystal path and a virtual beam indicator on the live display. As illustrated in Fig. 6 ▸, this overlay allows the user to verify that the beam consistently follows the crystal’s path, ensuring optimal alignment during diffraction data acquisition.

The ‘Update Trajectory’ button adds significant flexibility to the crystal tracking workflow. By re-displaying the previously acquired images from the ‘Record’ step, this feature allows users to replace or fine-tune the trajectory map as part of the setup before beginning a new 3D-ED acquisition, either by adjusting it for different regions of the same crystal or by defining a new path for another crystal that remained within the field of view during the angular range. For instance, in the images illustrated in Fig. 5 ▸, more than six different crystals remained within the field of view for the given angular range. Using the ‘Update Trajectory’ feature, we were able to collect 3D-ED data sets on all six crystals without needing to repeat the ‘Record’ step. This not only significantly improves the efficiency of data acquisition but also minimizes additional beam exposure. We must point out that this method of crystal tracking relies on the reproducibility of the crystal movement in consecutive rotation cycles. If the mechanical instabilities of the goniometer have a random character this method cannot be applied. In our TEM, a recorded trajectory can be reused more than ten times with negligible deviation. This stability and reproducibility allowed us to consistently track nanoparticles between 50 and 100 nm in size using *LibraEDT* (Cordero Oyonarte *et al.*, 2025 ▸).

### 3.5. Serial electron diffraction

Section 5 of the interface is dedicated to the acquisition of serial ED data. The data are acquired by scanning the stage in a manner similar to fixed-target serial crystallography (Hunter *et al.*, 2014 ▸). The serial ED approach, as implemented in *LibraEDT*, has been successfully employed for the solution of crystal structures. Comprehensive details regarding the methodology and technical implementation will be provided in a forthcoming publication.

### 3.6. Detector control

Section 6 of the interface is the detector control where *LibraEDT* manages data acquisition settings and serves as a bridge between the Client and Server Modules.

**Figure 7:**
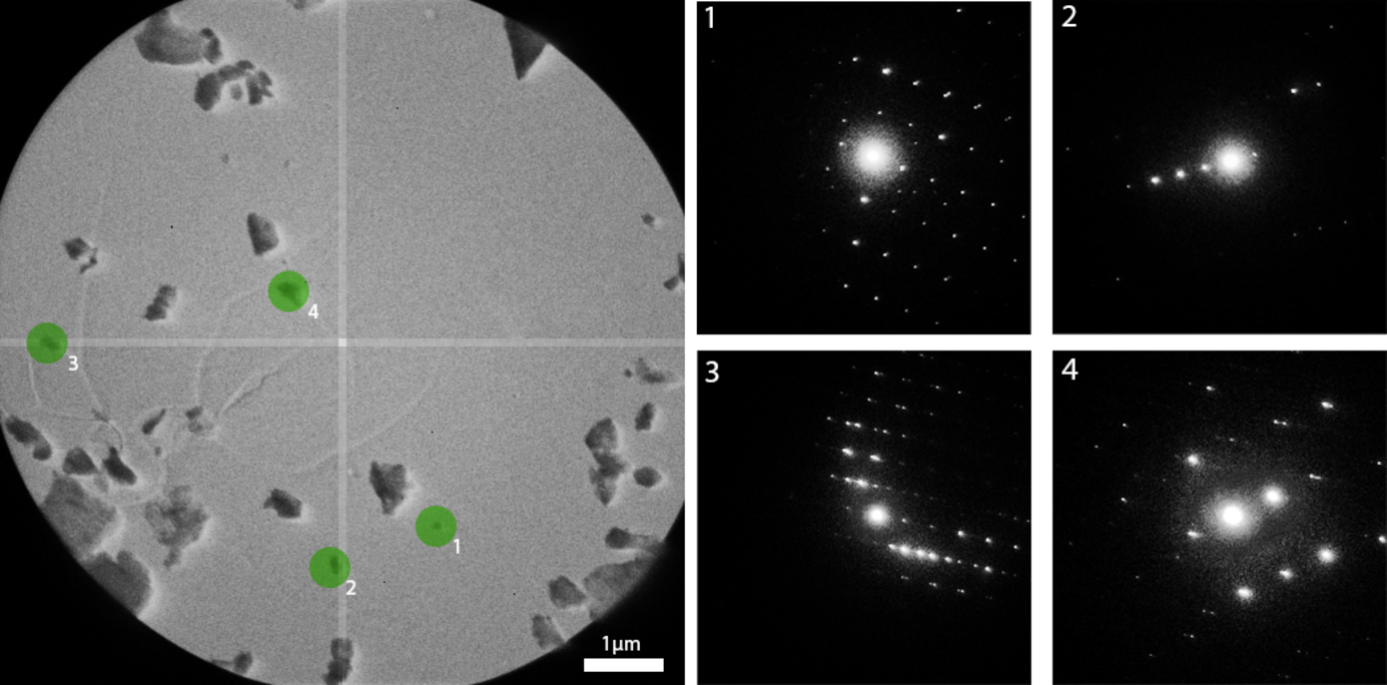
Interactive window in which the user can select several positions and automatically assess the diffraction quality.

One of the primary functions of this section is to send requests to the server, such as initiating image acquisition or adjusting the exposure time. For example, the ‘Save Raw Img’ button sends a request to capture a single image from the detector and store it on disk. In contrast, the ‘Start Streaming’ button requests the server to acquire images continuously from the detector and transmit them to the client. These images are displayed in real time on an interactive window, providing a live view of the sample. A useful feature of this interactive window is the ability to assess a diffraction pattern for one or multiple crystals directly from the window before starting the data acquisition process. If the user double clicks on any crystal position, *LibraEDT* then loads the corresponding diffraction settings (section 2 of the interface, Section 3.2.2) and shifts the beam to the first location using the calibrated beam shift information (section 2 of the interface, Section 3.2.1), immediately displaying the pattern. If several positions have been selected, the user can scroll the mouse wheel to move the beam sequentially between crystals without having to return to real space (Fig. 7 ▸). When diffraction patterns are displayed, the user can visualize resolution rings and reflection peaks by enabling the ‘Rings’ and ‘Peaks’ checkboxes.

Additionally, all image manipulation and post-processing, such as drawing resolution rings or highlighting peaks or gain and brightness adjustments, are performed on the client side using the *OpenCV* library (Bradski, 2000 ▸).

To ensure efficient data organization during diffraction data acquisition, each data set is systematically stored within a hierarchical folder structure following the pattern Operator­Name/SampleName/Date/CrystalNumber. When data are collected from additional crystals, the CrystalNumber increments automatically, ensuring that each new data set is saved in the next sequential folder (CrystalNumber+1).

### 3.7. Corrections and data preparation in the background

In addition to its user-controlled features, *LibraEDT* performs several processes in the background to enhance data quality. For instance, flat-field correction and dead-pixel correction are applied automatically to all acquired images, improving the accuracy of the diffraction patterns and reducing artefacts. The software also keeps track of the metadata associated with each data set, including acquisition parameters, timestamps and beam settings. These metadata are embedded directly into the saved data files, ensuring reproducibility of the experimental conditions. Additionally, the crystal tracking trajectory is saved to disk, providing a record of the crystal’s movement during the experiment.

Furthermore, *LibraEDT* automatically prepares input files compatible with widely used data processing software such as *PETS2* (Palatinus *et al.*, 2019 ▸), *XDS* (Kabsch, 2010 ▸) and *DIALS* (Winter *et al.*, 2018 ▸). This enables automated processing of the collected data, giving users the possibility of solving the crystal structure within minutes after data acquisition.

All GUI-adjusted parameters are consistently saved to disk. When the software is reopened by the same user or another, *LibraEDT* automatically reloads these settings, provided they were saved on the same day.

## 4. Workflow for data acquisition using *LibraEDT*

**Figure 8:**
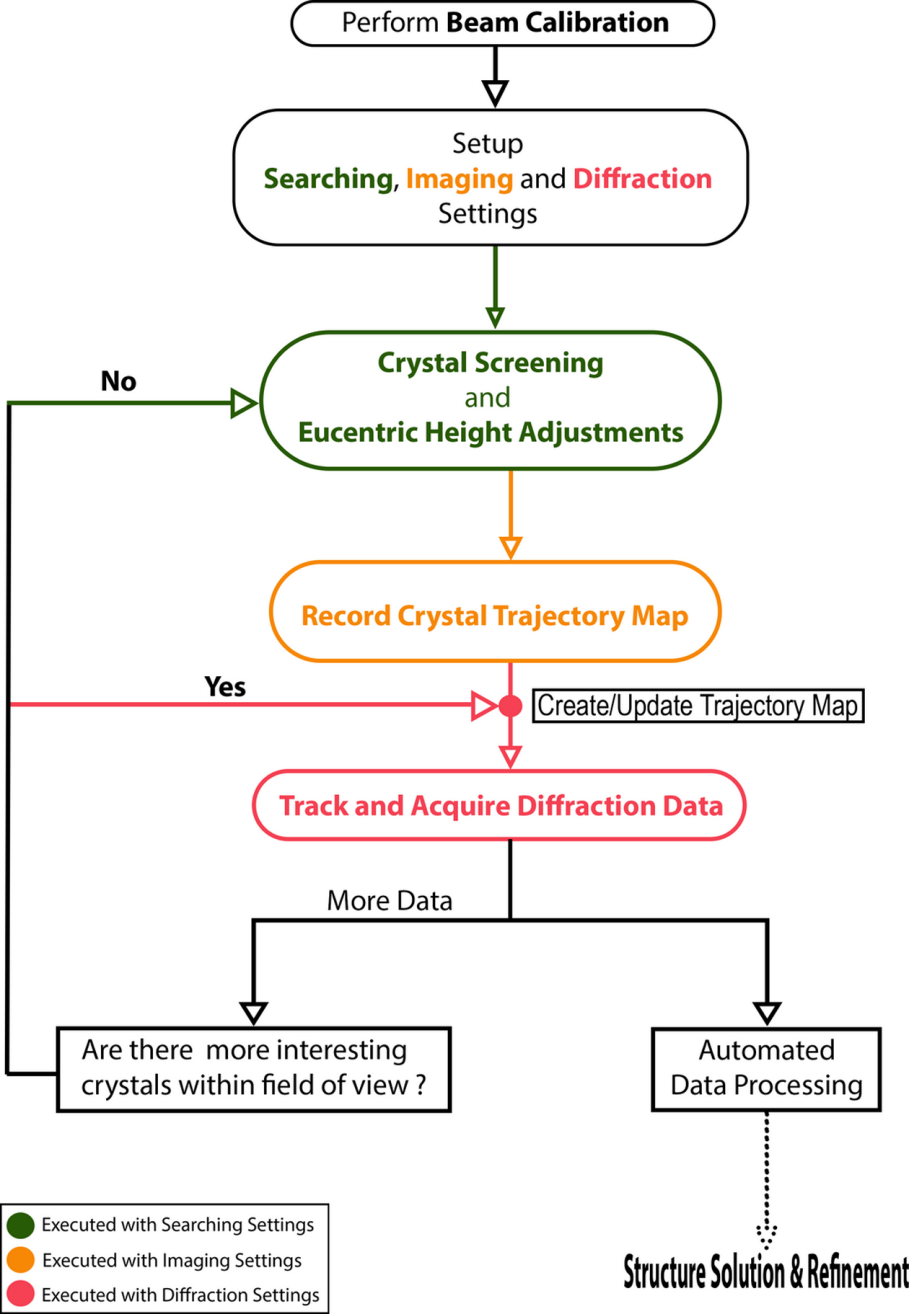
cRED/PEDT data acquisition workflow using *LibraEDT*.

The overall workflow for data acquisition using *LibraEDT* is summarized in Fig. 8 ▸. The process begins with beam calibration to ensure accurate beam positioning, followed by setting up the searching, imaging and diffraction settings. After configuration, the operator proceeds to crystal screening and eucentric height adjustments to identify suitable crystals and align them for diffraction. If a suitable crystal is found, the next step is to record the crystal trajectory map, which defines the crystal’s movement during stage rotation. The recorded trajectory is then used in the ‘Track & Acquire’ diffraction data step, where diffraction patterns are collected while dynamically tracking the crystal’s position. Once the data collection is complete, the corresponding data set can be automatically processed, as the input files for software such as *PETS2*, *XDS* and *DIALS* are already prepared. On the other hand, the user may acquire additional data by reusing the recorded images to update the trajectory map for another crystal within the field of view, or they can move to a different stage position to restart the crystal screening and eucentric height adjustment steps.

## 5. Case studies

### 5.1. Lamotrigine, a known organic crystal

Our first example concerns the structure determination of a beam-sensitive organic compound, lamotrigine, purchased from Sigma Aldrich. Lamotrigine, 6-(2,3-dichlorophenyl)-1,2,4-triazine-3,5-diamine, is a pharmaceutical compound used to treat epilepsy and bipolar disorder. The compound crystallizes in monoclinic space group *C*2/*c* and its crystal structure was determined relatively recently (Sridhar & Ravikumar, 2009 ▸).

#### 5.1.1. Sample preparation and data acquisition for lamotrigine

**Figure 9:**
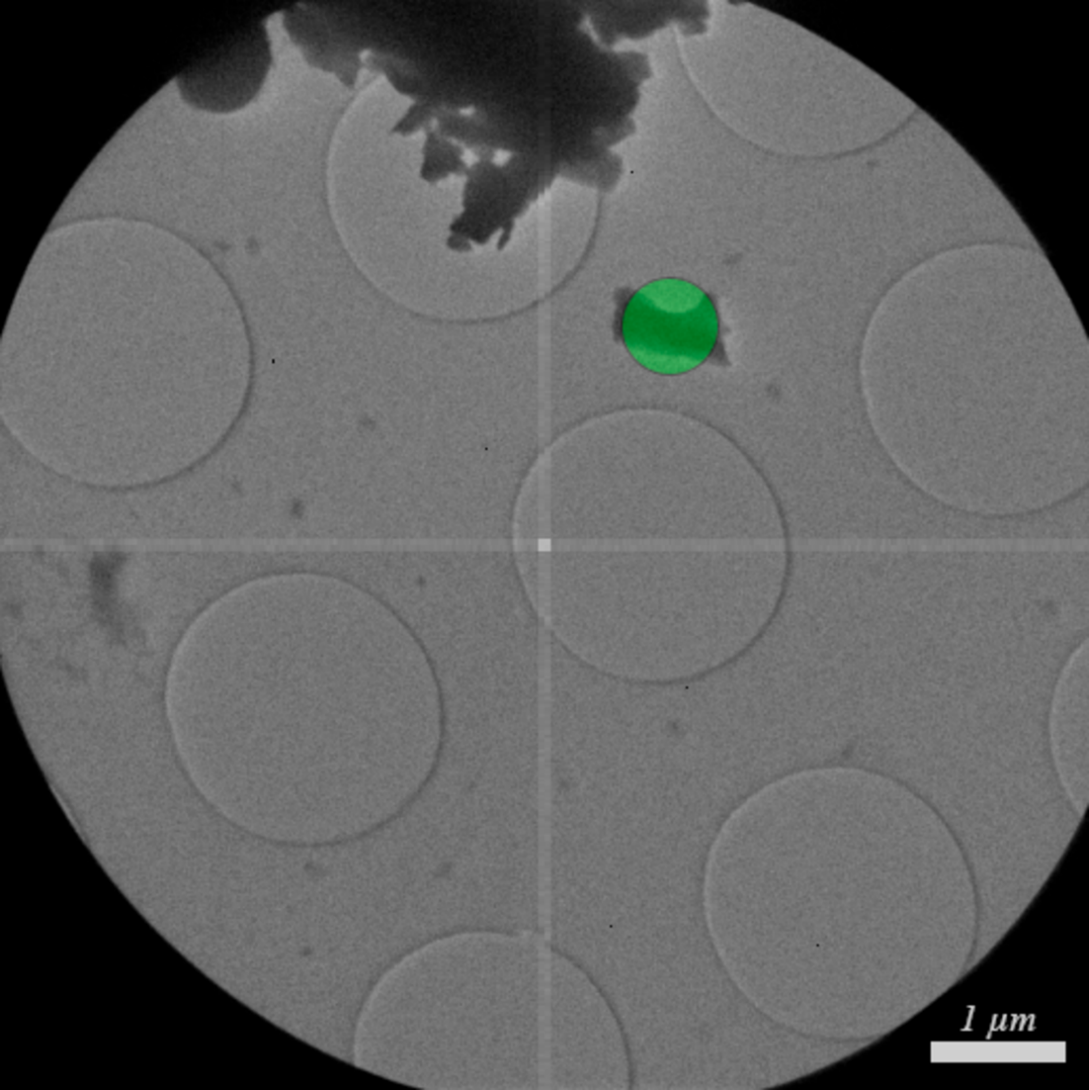
Image of the lamotrigine crystal selected for data collection. The green overlay represents the approximate size of the electron beam used during diffraction, which was set to a diameter of approximately 550 nm.

The lamotrigine crystals were prepared by crushing the powder between two glass slides and placing the crystals on a Quantifoil TEM grid. The electron microscope was operated at an accelerating voltage of 120 kV. For the searching and imaging settings, an aperture of 300 µm was used, resulting in a beam size of approximately 9 µm with a convergence semi-angle of 5 µrad, which corresponds to the minimal electron flux available in the microscope for a given filament current. The images were acquired every 2 s with an exposure time of 50 ms. For the diffraction settings, the convergence semi-angle was increased to 40 µrad and a condenser aperture of 19 µm was used, producing a beam size of approximately 550 nm (Fig. 9 ▸ and Table 1 ▸). The experiment was performed with a rotation speed of 2.00° per second and an exposure of 500 ms per frame, covering 120.5° of reciprocal space in a total time of 60.43 s. The beam stayed on the crystal for the entire rotation.

#### 5.1.2. Data analysis and structure solution for lamotrigine

Data analysis for lamotrigine was conducted using *XDS*, starting from the .inp file generated by *LibraEDT*. Indexing was successfully performed, resulting in a monoclinic unit cell with parameters *a* = 20.081 (4) Å, *b* = 8.8640 (18) Å, *c* = 14.091 (3) Å, α = 90.000°, β = 109.48 (3)° and γ = 90.000°, and the space group *C*2/*c*. The cut-off resolution, defined on the baseis of *CC*_1/2_, was set to 0.85 Å. The internal *R* factor (*R*_int_) was 0.103, indicating high-quality ED data. Structure solution was then achieved using *SHELXT* (Sheldrick, 2015*a* ▸), where all non-hydrogen atoms were successfully localized in the initial solution.

#### 5.1.3. Structure refinement for lamotrigine

**Figure 10:**
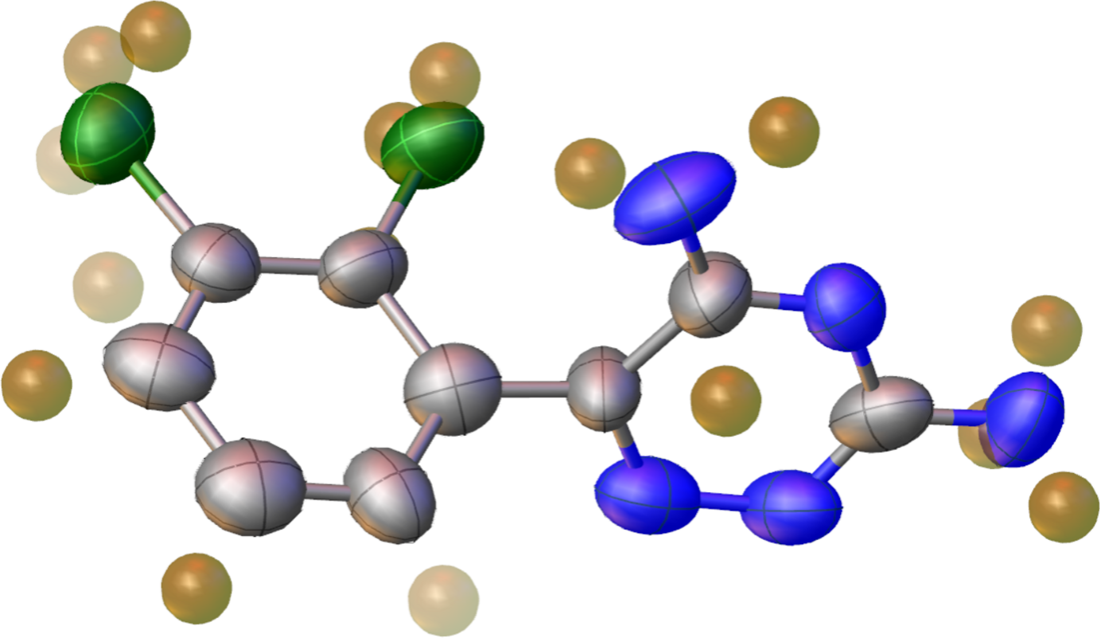
Kinematical refinement of lamotrigine, with all hydrogen positions clearly identified, as indicated by the strong *Q*-peaks in brown. Green denotes chlorine, grey carbon and blue nitrogen.

The structure was refined using the kinematical approximation with *SHELXL* (Sheldrick, 2015*b* ▸), run through *OLEX2* (Dolomanov *et al.*, 2009 ▸). The refinement was carried out without applying any restraints on bond lengths, bond angles or ring planarity. All atomic displacement parameters (ADPs) were refined anisotropically. The process converged with a final *R*_1_ value of 0.1754. Notably, all hydrogen atoms were clearly visible in the difference Fourier map and could be unambiguously placed, as shown in Fig. 10 ▸. The final crystallographic data are detailed in Table 2 ▸. The main difference compared with the reference X-ray structure (CSD refcode EFEMUX01; Sridhar & Ravikumar, 2009 ▸) lies in the unit-cell parameters, which differ by approximately 3% in the 3D-ED data. Such deviations are commonly observed in ED experiments and may result from factors such as inaccurate calibration of the pixel size, distortions introduced by the microscope’s magnetic lenses (Brázda *et al.*, 2022 ▸) or the high-vacuum conditions during data collection, which can lead to expansion or contraction of the unit cell. Additionally, the ADPs refined from the 3D-ED data are generally higher than those reported in the reference X-ray structure, with average values of 0.08 and 0.04 Å^2^ for the 3D-ED and X-ray models, respectively. This may result from the use of the kinematical approximation, which does not account for multiple scattering and may overestimate thermal motion or the thermal effect induced locally by the electron beam. The similarity between the two structures, without considering the hydrogen atoms, was further evaluated using the structural comparison tool in *Compstru* (de la Flor *et al.*, 2016 ▸), which computes a similarity metric δ based on differences in atomic positions weighted by site multiplicity as well as the ratios of the corresponding lattice parameters. In this case, the similarity measure δ was found to be 0.037 with an average deviation of the atomic positions of 0.034 Å, indicating a high degree of structural agreement despite the variation in unit-cell dimensions. A full dynamical refinement of this data set would likely result in more accurate ADPs and bring the bond lengths and angles closer to those observed in the reference model.

### 5.2. PZMOF, a zinc and protocatechuic-acid based metal–organic framework

Our next example presents the crystallographic analysis of a novel metal–organic framework (MOF), (C_21_H_10_O_13_Zn_4_)_*n*_·0.47H_2_O, PZMOF, synthesized in our laboratory.

#### 5.2.1. Preparation of PZMOF

3,4-Dihydroxybenzoic acid (protocatechuic acid, 2.00 mmol) and NaOH (2.00 mmol) were dissolved in de­ionized water (10.0 ml). In a separate flask, anhydrous Zn(OAc)_2_ (2.00 mmol) was dissolved in deionized water (10.0 ml). Under vigorous stirring at room temperature, the zinc solution was added dropwise to the ligand solution, upon which a green precipitate formed immediately. The mixture was stirred for an additional 4 h to ensure complete reaction. The product was then collected by filtration, washed with acetone and left to dry overnight under ambient conditions.

#### 5.2.2. Data acquisition for PZMOF

**Figure 11:**
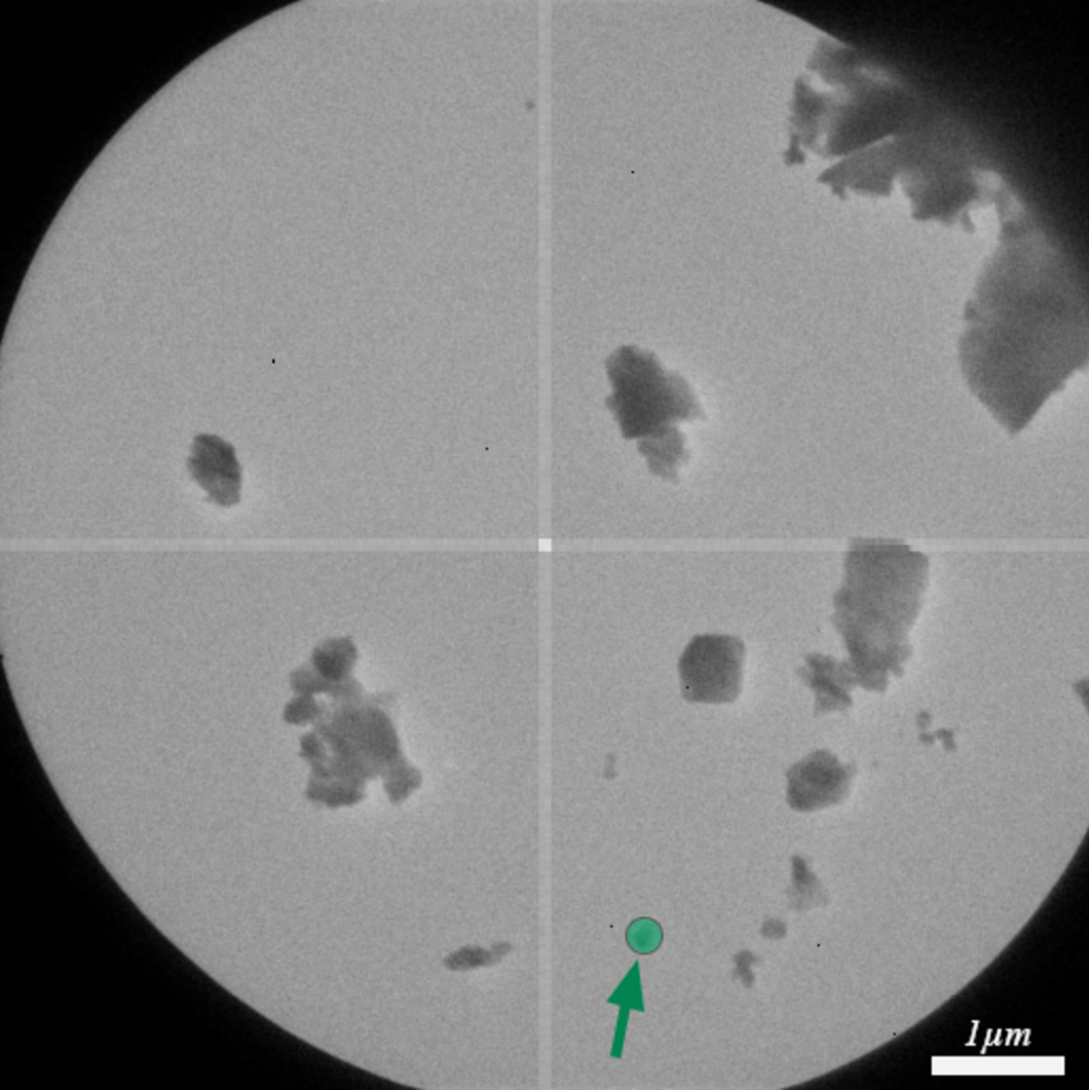
Image of the PZMOF crystal selected for data collection. The green overlay represents the approximate size of the electron beam used during diffraction, which was set to a diameter of approximately 250 nm.

The PZMOF crystals were prepared by placing a dilute suspension onto a continuous carbon grid and allowing it to dry under ambient conditions. The data acquisition was performed under conditions similar to those used for lamotrigine, with the following key differences: a condenser aperture of 10 µm was used, resulting in a beam size of approximately 250 nm at the sample level. The rotation speed was set to 2.00° per second and a total of eight data sets were collected, each covering a tilt range from −60° to 60° over 60 s. All other parameters, including low-fluence techniques during the searching and imaging stages, were consistent with those employed for lamotrigine. The experimental parameters of PZMOF are detailed in Table 3 ▸, and a crystal of approximately 100 nm in size used for data acquisition is shown in Fig. 11 ▸.

#### 5.2.3. Data analysis and structure solution for PZMOF

The different data were processed using *PETS2.0* and the automatically generated input file. The best data resulted in 98.12% of successful indexing of the retrieved diffraction peaks with a triclinic unit cell, *a* = 8.871 (2) Å, *b* = 13.045 (3) Å, *c* = 13.150 (3) Å, α = 65.67 (3)°, β = 70.86 (3)° and γ = 81.57 (3)°, and a unit-cell volume of 1309.8 (6) Å^3^. The reflections were then integrated and scaled to account for experimental factors. The scaling process revealed an *R*_int_ value of 0.104 for the observed data and 0.1871 for all reflections, with an average redundancy of 1.89, which is considered reasonable for ED data. The completeness of the data was 68%. To prepare for dynamical refinement, a dedicated .hkl file with extension .cif_pets is generated by preparing overlapping virtual frames, making sure that reflections are properly integrated (Klar *et al.*, 2023 ▸). Structure solution was obtained in space group 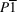 using *SHELXT* (Sheldrick, 2015*a* ▸) with all non-hydrogen atoms successfully localized.

#### 5.2.4. Structure refinement for PZMOF

The structure of PZMOF was refined in two stages: using kinematical refinement to develop a reasonable starting model, followed by dynamical refinement to achieve a more accurate representation of the crystal structure. The initial refinement of the PZMOF structure was conducted using the *JANA* software (Petříček *et al.*, 2014 ▸; Petříček *et al.*, 2023 ▸) employing a kinematical approach, with restraints applied only to the aromatic rings of the linker to maintain planarity and equal bond lengths. The refinement yielded an *R*_1_ value of 0.2997, which provided a sufficiently accurate model to proceed to the next stage. Following the kinematical refinement, the structure was further refined using a dynamical approach, also implemented in *JANA*. The parameters used for dynamical refinement are listed in Table 4 ▸. To preserve a reasonable data-to-parameter ratio, all atoms were refined with isotropic displacement parameters. After several cycles of dynamical refinement, the *R*_1_ value improved to 0.1164, indicating a much better fit to the experimental data. This substantial reduction in the *R*_1_ value reflects the increased accuracy of the model, particularly in terms of atomic positions and displacement parameters.

**Figure 12:**
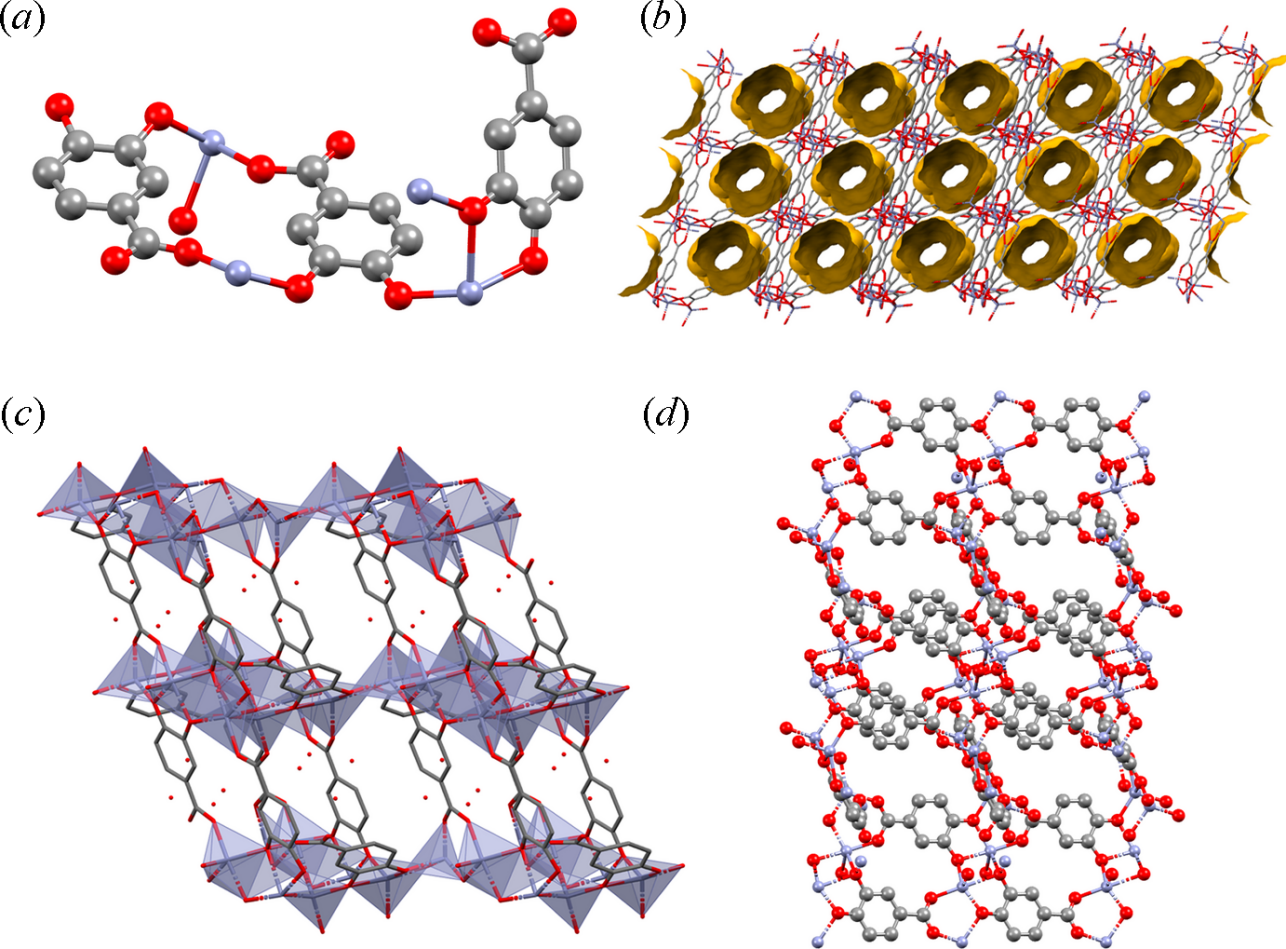
Structural representations of PZMOF. (*a*) Asymmetric unit showing four zinc atoms and three protocatechuic acid molecules. (*b*) Unit cell viewed along the *a* axis, with voids occupying approximately 27% of the unit-cell volume, depicted in yellow. (*c*) View along the *b* axis showing the water molecules in the channels and the different coordination geometry of the zinc atoms, represented as polyhedra. (*d*) View along the *c* axis, illustrating the overall framework structure. Hydrogen atoms have been omitted for clarity.

PZMOF exhibits rhombic channels extending along the 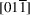 crystallographic direction. The channel walls are constructed from protocatechuate ligands, while chains of Zn—O polyhedra, one strongly elongated octahedron, two distorted square pyramids and one tetrahedron, bridge into four-membered rings that act as the channel hinges. Within the channels of the MOF, two water sites are located, O5 co­ordinating weakly to Zn1 and O*W*1 situated close to the centre of the channel. Both show relatively large ADPs, indicative of disorder and partial occupancy. Their site occupancies were therefore freely refined, resulting in values of 0.473 and 0.651, respectively. Of the three protocatechuate ligands in the asymmetric unit, two are fully deprotonated and bind through all four oxygen donors. Interestingly, the third ligand retains its carboxyl H atom. One carboxyl oxygen (O9) coordinates to Zn2, while the other (O8), still retaining the H atom, remains uncoordinated. Charge-balance calculations supported this assignment by showing that the unit cell carries a net charge of −1 without the carboxyl H atom, whereas including the H atom restores neutrality. Void analysis using the *Mercury* software (Macrae *et al.*, 2020 ▸) indicates a total porosity of 27% (Fig. 12 ▸).

## 6. Conclusions

In this work, we have introduced the *LibraEDT* software, developed to optimize and automate 3D-ED experiments on the Zeiss Libra 120 kV microscope. The software’s capabilities in maintaining precise crystal positioning, automating data acquisition and applying necessary corrections have improved the accuracy and efficiency of data collection, particularly for nanocrystals and other challenging materials. The successful characterization of lamotrigine and PZMOF demonstrates the practical utility of *LibraEDT* in real-world applications.

The object-oriented programming (OOP) design of *LibraEDT* provides a flexible foundation that could be adapted for use with other instruments.

*LibraEDT* contributes to the ongoing development of 3D-ED methodologies by offering a practical solution that speeds up the screening of new unknown powder samples, which is the first step towards a high-throughput system for 3D-ED. Using *LibraEDT* it is possible to screen dozens of crystals in a single TEM session while simultaneously analysing the data, which provides useful preliminary insights into the sample’s structural characteristics in real time. Moreover, maximizing the angular range during data collection, even when illuminating a small area, thanks to the use of crystal tracking, significantly improves the accuracy of 3D-ED structural characterizations, particularly for beam-sensitive materials.

## Supplementary Material

Crystal structure: contains datablock(s) global, PZMOF, lamotrigine_3ded. DOI: 10.1107/S1600576725006892/te5151sup1.cif

Structure factors: contains datablock(s) lamotrigine_3ded. DOI: 10.1107/S1600576725006892/te5151lamotrigine_3dedsup2.hkl

CCDC references: 2445870, 2445871

## Figures and Tables

**Table 1:** Experimental 3D-ED acquisition parameters for lamotrigine

Parameter	Value
Wavelength (Å)	0.0335
Tilt range (°)	121
Rotation speed (° s^−1^)	2.00
Exposure time (ms)	500
Oscillation range (° per frame)	1.04
No. of frames	116
Condenser aperture (µm)	19
Beam size (nm)	550
Convergence semi-angle (µrad)	40
Fluence (e^−^ Å^−2^)	0.6

**Table 2:** Crystallographic data for lamotrigine

Crystal data	
Chemical formula	C_9_H_7_Cl_2_N_5_
*M*_r_	259.12
Crystal system, space group	Monoclinic, *C*2/*c*
Temperature (K)	293
*a*, *b*, *c* (Å)	20.081 (4), 8.8640 (18), 14.091 (3)
β (°)	109.48 (3)
*V* (Å^3^)	2364.6 (9)
*Z*	8
Radiation type	Electrons, λ = 0.0335 Å
No. of measured, independent, observed reflections	4718, 1852, 923 [*I* > 2σ(*I*)]
*R*_int_	0.103
Completeness	92.7%
(sinθ/λ)_max_ (Å^−1^)	0.585

Kinematical refinement statistics	
*R* [*F*^2^ > 2σ(*F*^2^)], *wR*(*F*^2^), *S*	0.184, 0.498, 1.51
No. of reflections	1852
No. of parameters	148
No. of restraints	0
Δρ_max_, Δρ_min_ (e Å^−3^)	0.17, −0.22
Similarity (δ)	0.037
Average deviation (Å)	0.034

**Table 3:** Experimental 3D-ED acquisition parameters for PZMOF

Parameter	Value
Wavelength (Å)	0.0335
Tilt range (°)	121
Rotation speed (° s^−1^)	2.00
Exposure time (ms)	500
Oscillation range (° per frame)	1.04
No. of frames	116
Condenser aperture (µm)	10
Beam size (nm)	300
Convergence semi-angle (µrad)	40
Fluence (e^−^ Å^−2^)	0.6

**Table 4:** Crystallographic data for PZMOF

Crystal data	
Chemical formula	C_21_H_9_O_13.6727_Zn_4_
*M*_r_	741.6
Crystal system, space group	Triclinic, 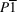
Temperature (K)	293
*a*, *b*, *c* (Å)	8.871 (2), 13.045 (3), 13.150 (3)
α, β, γ (°)	65.67 (3), 70.86 (3), 81.57 (3)
*V* (Å^3^)	1309.8 (6)
*Z*	2
Radiation type	Electrons, λ = 0.0335 Å
No. of measured, independent, observed reflections	32739, 3532, 979 [*I* > 3σ(*I*)]
*R*_int_	0.104
Completeness	68%
(sinθ/λ)_max_ (Å^−1^)	0.620

Dynamical refinement statistics and parameters	
*R* [*F* > 2σ(*F*)], *wR*(*F*), *S*	0.116, 0.130, 1.76
No. of reflections	3532
No. of parameters	173
No. of restraints	34
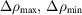 (e Å^−3^)	0.53, −0.54
Max. diffraction vector *g*_max_	1.44
Max. excitation error (matrix step)	0.01
Max. excitation error (refinement step)	0.10
*RS*_*g*_(max)	0.66
SCA diameter	10
*DS*_*g*_(min)	0
No. of integration steps	100

## Data Availability

The source code of the software discussed in this paper is freely available on Github, https://github.com/Thioo/LibraEDT_Client and https://github.com/Thioo/LibraEDT_Timepix_Server, while the 3D-ED data sets used in this study are available from the NanED community at Zenodo, https://zenodo.org/records/15269010. The crystal structures of lamotrigine and PZMOF have been deposited with the Cambridge Structural Database under deposition numbers 2445870 and 2445871, respectively.
